# Frontiers in Plant Breeding: Perspectives for the Selection of Vegetables Less Susceptible to Enteric Pathogens

**DOI:** 10.3389/fmicb.2020.01087

**Published:** 2020-05-28

**Authors:** Tania Henriquez, Anna Lenzi, Ada Baldi, Massimiliano Marvasi

**Affiliations:** ^1^Biozentrum, Ludwig-Maximilians-Universität München, Munich, Germany; ^2^Department of Agriculture, Food, Environment and Forestry (DAGRI), University of Florence, Florence, Italy; ^3^Department of Biology, University of Florence, Florence, Italy

**Keywords:** plant breeding, enteric pathogens, biomarkers, cultivars, produce, food safety

## Abstract

Fresh vegetables including baby greens, microgreens, and sprouts can host human pathogens without exhibiting any visible signs of spoilage. It is clear that the vast majority of foodborne disease outbreaks associated with vegetable produce are not simply a result of an oversight by a producer, as it was shown that zoonotic pathogens from *Enterobacteriaceae* can contaminate produce through various routes throughout the entire production cycle. In this context, phenotypic and genotypic signatures have been used since early ages in agriculture to obtain better produce, and can be used today as a strategy to reduce the risk of outbreaks through plant breeding. In this mini-review, we provide an updated view and perspectives on to what extent the selection of biological markers can be used to select safer cultivars of vegetable crops such as tomato (the most studied), leafy greens and cabbage. Once this knowledge will be better consolidated, these approaches should be integrated into the development of comprehensive farm-to-fork produce safety programs.

## Human Pathogens in Crop Production Environment

**FIGURE 1:**
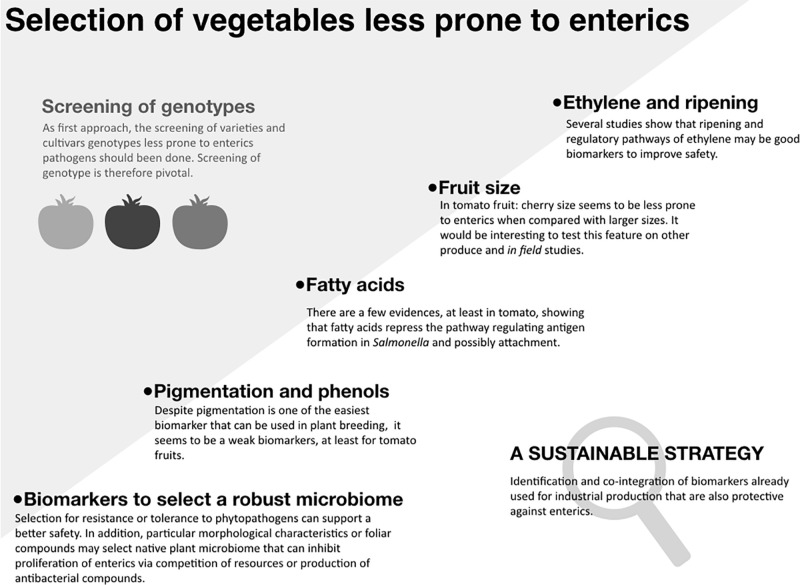
Selection of vegetables less prone to enteric pathogens. “Screening of genotypes”: The gray shadow panel shows that within same species there is a significant variability to the proliferation of enterics. Such different susceptibility can be the result of specific genetic traits that can be inherited by the offspring. The diagram shows the current advances in this direction, speculating which biomarkers may be the most promising. “Sustainable strategy” panel: To give an example, in tomato, some of the ripening related genes that are already used to increase shelf life could be used to develop vegetables less prone to enteric pathogens (Bai and Lindhout, 2007).

Outbreaks linked to the consumption of fresh fruits, vegetables, and sprouts suggest that human pathogens can contaminate produce pre- and/or post-harvest (Bartz et al., 2014). Human enteric pathogens, such as non-typhoidal *Salmonella* and Shiga toxin-producing *Escherichia coli* can survive in the crop production environment, causing recurrent outbreaks (U.S. FDA, 2019). The majority of outbreaks of gastrointestinal illnesses have been associated with fruits, lettuce, alfalfa sprouts, spinach and tomatoes (Dewey-Mattia et al., 2018; CDC, 2019). Based on these observations, it is reasonable to hypothesize that enteric pathogens interact differently with various crops (although a number of other hypotheses can be offered and tested). Such pathogens also exhibit different contamination in greenhouses or in the field (Barak et al., 2011; Barak and Schroeder, 2012). These pathogens can survive in soil and water for extended periods of time and surface irrigation water improperly treated has been commonly identified as a source of contamination (Lapidot and Yaron, 2009; Moorman et al., 2014). Good Agricultural Practices and agronomical operations, as well as intervention technologies optimized to manage plant pathogens and safety have been put in place to minimize the risk of produce contamination and the spread of the pathogens through the supply chain (Bartz et al., 2014; Marvasi et al., 2015a; Murray et al., 2017). In addition to these tools, plant breeding has been recently suggested as another opportunity to enhance produce safety. This mini-review focuses on the feasibility of harnessing crop’s genetic potential to improve produce safety, with the emphasis on enteric pathogenic bacteria, primarily non-typhoidal *Salmonella* and Shiga toxin-producing *E. coli*, which have been the primary culprits of a large number of outbreaks linked to vegetable produce. In the first section of this mini-review we show the intra-species variability in the susceptibility to contamination and proliferation of enteric pathogens. Such variability among varieties and cultivars can be the result of specific genomic traits that can be transferred to the offspring. In the second section, we explore cases when potential biomarkers are identified and tentatively associated with the response to enteric pathogens (Figure 1).

## Cultivars of Vegetable Crops Differ in Their Susceptibility to Enteric Pathogens

### Tomato

Tomato is by far the most studied species, probably due to the fact that it is the most important vegetable crop worldwide, and also often involved in enteric pathogens outbreaks. A greenhouse screening of 31 cultivars with characteristics that could conceivably affect how conducive tomatoes are to *Salmonella* proliferation has been carried out (Marvasi et al., 2014a). From this screening, tomato cultivar ‘Sun Gold’ and ‘Snow White’ were shown to be less prone to support *Salmonella* proliferation. This study also showed that cherry tomatoes (small size) were generally less conducive to proliferation of *Salmonella* when compared with tomato cultivars with a regular size (Marvasi et al., 2014a). A few studies were also carried out under field conditions. The post-harvest proliferation of *Salmonella* in the post-harvest tomato cultivars ‘Bonny Best,’ ‘Florida-47,’ and ‘Solar Fire’ grown under a variety of irrigation regimes showed that ‘Bonny Best’ was significantly less prone to *Salmonella* proliferation when compared with ‘Solar Fire’ but not when compared with ‘Florida-47’ (considering all maturity stages) (Marvasi et al., 2013a). Interestingly, in the greenhouse study, unripe ‘Bonny Best’ tomato fruits were less susceptible to *Salmonella* also when compared with ‘Florida-47’ (Marvasi et al., 2014a). This suggests that caution should be taken against directly translating the results of greenhouse studies to field conditions. The cultivar ‘Solar Fire’ was not significantly different from ‘Sebring’ when subjected to different fertilization regimes (Marvasi et al., 2013a, 2014b).

In another field study, 13 tomato cultivars selected based on a range of distinct fruit phenotypes, including morphology, pigmentation and resistance to phytopathogens were tested for susceptibility to *Salmonella* surface colonization (Han and Micallef, 2014). The study demonstrated that fruits and leaves of the same cultivar differed in their ability to suppress or support *Salmonella* growth arguing for the important role of tissue differentiation (Han and Micallef, 2014). Fruits of cultivar ‘Heinz-1706’ were the least colonized by *Salmonella* Newport, while the highest populations were observed on ‘Nyagous.’ By contrast, seedlings of the cultivar ‘Florida-91’ supported lowest populations of *Salmonella* Newport while the cherry variety ‘Virginia Sweets’ supported the highest. For seedling leaves infected with *Salmonella* Typhimurium the lowest susceptible was the cultivar ‘Nyagous’ and the highest were the cultivars ‘Heinz-1706’ and ‘Moneymaker’ (Han and Micallef, 2014). The comparative genomics of the pathogenic strains and in particular different serovars could help to determine why they behave differently during infection (e.g., *Salmonella* Typhimurium versus Newport) (Teplitski and de Moraes, 2018). Although tomato leaves are not edible, the information about *Salmonella* attachment and susceptibility to leaf colonization are relevant since *Salmonella* residing on leaves can be transmitted to fruits (Han and Micallef, 2014).

### Cabbage

Response of cabbage cultivars to internalization or surface survival of *Salmonella* and *Escherichia coli* O157:H7 was studied by Erickson et al. (2019a;b). In a growth chamber study, internalized *Salmonella* was detected in cabbage within 24 h with prevalence ranging from 62% plants for the cultivar ‘Super Red 80’ to 92% for ‘Red Dynasty.’ The study showed that surface survival of both *Salmonella* and *Escherichia coli* O157:H7 on small cabbage plants over nine days was significantly affected by cultivars: pathogens survived the most on the cultivar “Farao” (Erickson et al., 2019b). In a field study which compared medium-sized cabbage heads, ‘Red Dinasty’ was more likely to be positive for *Salmonella* and *E. coli* O157:H7 (3.0 and 11.5 times, respectively) on day 5 post-inoculation when compared with the cultivar ‘Bravo F1’ (Erickson et al., 2019a).

### Leafy Greens

When seeds of cilantro, parsley, radicchio, endive, lettuce and spinach were sown in the same pots containing contaminated soil, radicchio and endive had a significantly higher contamination index (CI) than lettuce (Barak et al., 2008). Since radicchio, endive, and lettuce all belong to the *Asteraceae*, these results revealed different response to enteric pathogens within the same Family, suggesting a possible effect of the genotype, at least under these greenhouse conditions (Barak et al., 2008). It must be mentioned that over the last decade, lettuce was instead associated with the highest number of outbreak investigations (U.S. FDA, 2019), but this could be due to the much higher acreage and consumption of lettuce versus other leafy crops (USDA - Statistics, 2019). The experiments described above were conducted in greenhouses and therefore may have missed some typical conditions of the field environment. Other field studies were carried out aiming to compare attachment of *Salmonella* on different lettuce cultivars. In this scenario prevalence of contamination was observed on leaves with the following order (from the less to the most): ‘Muir’ < ‘Gabriella’ < ‘Green Star’ = ‘New Red Fire’ < ‘Coastal Star’ (Erickson et al., 2019c).

## Potential Biomarkers That Can Contribute to Produce Safety

We have previously seen that cultivars differ in their ability to support proliferation of enteric pathogens, therefore a number of potential biomarkers to be used in breeding programs can be obtained from studying such differences. Tomato is mainly used as a model plant for this purpose, but limited literature is also available on other raw eaten crops.

### Flavonoids, Carotenoids, and Phenolics

Pigmentation, due to the presence of flavonoids and carotenoids, is the easiest biomarker that can be used in plant breeding programs. A recent study tested whether pigmentation can serve as an appropriate indicator of plant susceptibility to human pathogens. Thirty-one different tomato cultivars, including those that show different pigmentations have been screened. Despite color differences, pigmentation *per se* did not appear to account for the increased proliferation of *Salmonella* (Marvasi et al., 2014a). Follow-up studies demonstrated that tomato phenolics rutin, quercetin and kaempferol did not impact growth of *Salmonella* in rich laboratory media. Therefore, these three phenolics may not be useful as biomarkers for selection either (Marvasi et al., 2014a). The discovery of any association of pigmentation or other phenotypic characteristics (for example flavors) with enteric pathogens conductivity would be an easy phenotypic trait to be used. Less susceptible leafy greens or cabbage cultivars may be also potentially screened for pigments or phenolic compounds. To our knowledge no research has been carried out on these species.

### Fatty Acids

The presence of fatty acids has been proposed as a possible indicator of susceptibility to enteric pathogens. Linolenic and linoleic acids are unsaturated fatty acids present in tomato and are precursors of hexanal which contributes to the fruity flavor (Jadhav et al., 1972). Linoleic acid seems to be involved in proliferation of *Salmonella* (Noel et al., 2010; Marvasi et al., 2013b). Linoleic acid is a polyunsaturated omega-6 fatty acid and its accumulation is progressively reduced in mature tomato fruits (Carrari et al., 2006). Linoleic acid has been demonstrated to play a role in the regulation of *yihT* gene which is involved in the synthesis of the O-antigen capsule in *Salmonella*. It is therefore reasonable to assume that capsule may be involved in attachment/persistence also outside the human host (Maruzani et al., 2019). Interestingly, deletion of *yihT* in *S. enterica* sv. Typhimurium reduced competitive fitness of the pathogen in immature but not in ripe tomatoes, regardless of their color at maturity. Linoleic acid is able to completely repress the *yihT* gene in *Salmonella* and the suppression of such gene could reduce the proliferation in immature tomatoes. Repression of the *yihT* reporter was stronger in tomato cultivars ‘Kumato,’ ‘Snow White,’ and ‘Mariana’ when compared with the variety ‘Bloody Butcher’ (Marvasi et al., 2013b). However, the response seemed to be generic to other fatty acids because decanoic and linolenic also affected the *yihT* gene reporter (Noel et al., 2010; Marvasi et al., 2013b). Other studies showed that linoleic acid was responsible for the upregulation of *Salmonella fadH* gene in green tomato fruit (Noel et al., 2010). The *fadH* gene encodes 2,4-dienoyl-CoA reductase, an iron-sulfur flavoenzyme required for metabolism of unsaturated fatty acids with double bonds at even carbon positions (Hubbard et al., 2003). More studies should be conducted to determine if fatty acids could serve as biomarkers due to their ability to repress key genes of enteric pathogens. As per phenolic, to our knowledge no additional literature is available to associate fatty acids and susceptibility to enteric pathogens in other crops, such as leafy greens or cabbage.

### Ethylene

Ethylene is a plant hormone playing a key role in climacteric fruit ripening (Liu et al., 2015). *Salmonella* relies on a distinct set of metabolic and regulatory genes, which are differentially regulated *in planta* in response to the host genotype and during tomato fruit ripening (Noel et al., 2010). Because ethylene is involved in fruit ripening, it is clear that this metabolic pathway could be a good candidate to be further explored.

Fruits of three tomato cultivars, ‘Bonny Best,’ ‘Solar Fire,’ and ‘Florida-47,’ were harvested and infected with 100 CFU of *Salmonella* and incubated for a week at 22°C. In this experiment maturity stages of the fruits at the time of infection with *Salmonella* were assessed using the USDA tomato maturity chart. Maturity stages 5 and 6 at field harvest correspond to “ripe,” fruits that were harvested at 4 and 3 stages were considered “partially ripe,” and those that were harvested at 1 and 2 stages (and did not ripen beyond stage 5 during the experiment) were considered “unripe” (Marvasi et al., 2013a). The results showed that each ripening stage was significantly different in terms of *Salmonella* proliferation. The researchers found *Salmonella* counts to be at least 1 log higher in ripe tomatoes compared to the unripe tomatoes under the same growing conditions (Marvasi et al., 2013a). *Salmonella* ability to colonize bell peppers (cultivar ‘Aristotle’) at different maturity stages was also tested, showing that mature peppers were significantly more prone to support *Salmonella* when compared with the un-ripe ones (Marvasi et al., 2015b). The survival and/or proliferation of *Escherichia coli* O157:H7 and *Salmonella* on the surface of artificially bruised and unbruised tomatoes was tested at three ripeness stages (breaker, pink, and red) and two storage temperatures (10 and 20°C) (Tokarskyy et al., 2018). Tomatoes at the red ripeness stage showed a significant effect of bruising on *Salmonella* survival at both 10 and 20°C (Tokarskyy et al., 2018). Ripening is associated with tissue softening in tomato or bell peppers, therefore the proliferation of *Salmonella* during ripening may be associated with the ability of the bacterial cells to spread more easily into the softener tissue. In addition, a mature pericarp is also more prone to shallow wounds (Marvasi et al., 2014a). According with these results, it is reasonable to assume that testing a series of mutants in ethylene signaling could offer insights about the role of ethylene related genes and ripening during enteric pathogens proliferation. Results of the experiments with tomato mutants with specific defects in ethylene synthesis and perception suggested that ethylene signaling pathways mediated by RIN and NOR (MADS box and SPBP transcriptional factors, respectively) are more consequential that those that rely on the ethylene response sensor-like ethylene receptor NR (Fujisawa and Ito, 2013; Marvasi et al., 2014a). These mutants showed also changes in pigment production, being RIN and NOR not ripening (remaining green), while NR changing to dark red (Osorio et al., 2011; Liu et al., 2015). When post-harvest mature tomato fruits carrying RIN, NOR, and NR mutations were infected with *Salmonella*, reduced proliferation of *Salmonella* was observed when compared with the wild type parent ‘Ailsa Craig’ (Marvasi et al., 2014a). Some of these ripening related genes are already used in tomato breeding programs, therefore a systematic evaluation of the susceptibility of these cultivars to enteric pathogens can provide further information (Bai and Lindhout, 2007).

Another experiment to test the involvement of the ethylene cascade in the proliferations of enteric pathogens has been done by treating *Medicago truncatula* seedlings with the ethylene precursor 1-aminocyclopropane-1-carboxylic acid (ACC). The treatment strongly reduced endophytic populations of *Salmonella* (Iniguez et al., 2005). Are therefore ethylene mutants/variants good indicators? It is too early to advise, but the ethylene pathway may be a good candidate for further studies. In addition, the selection of plants that differently respond to the application of post-harvest ethylene can be interesting for produce safety (such as the ethylene receptor NR in tomato).

## Breeding Plants to Support a Robust Microbiome

Microbiome has been shown to contribute to the proliferation of *Salmonella* in tomato fruits (Marvasi et al., 2013a; Devleesschauwer et al., 2017). Tomato fruits hosting the native microbiome were less prone to *Salmonella* infection when compared with surface cleaned fruits (Marvasi et al., 2013a; Devleesschauwer et al., 2017). *Salmonella*, for example, seems to harbor traits allowing for its interaction with plants and its microbiota (Brandl et al., 2013). It is tempting to speculate that the selection for leaf morphology, presence of exudates or waxes can stimulate the robust “healthy” microbiome and discourage establishment of enteric pathogens. There are no specific studies in this area, however, a few publications support the overall rationale. First, it is well established that leaf architecture, nutrients and secondary metabolites impact the composition of the plant epibiome (Singh et al., 2019). Second, recent studies demonstrated that certain members of the native plant microbiome can inhibit proliferation of *Salmonella* and pathogenic *E. coli* (Brandl et al., 2013).

For example, an antagonist epiphyte to *Salmonella*, *Paenibacillus alvei TS-15*, was isolated from different plants native to the Virginia Eastern Shore tomato-growing region (Allard et al., 2014). The strain TS-15 exhibited broad-range antimicrobial activity against both major foodborne pathogens and major bacterial phytopathogens of tomato. Survival of *Salmonella* after inoculation was measured for groups with and without the antagonist at days 0, 1, 2, and 3 and either day 5 on blossoms or day 6 for fruits and leaves. After *P. alvei* strain TS-15 was applied onto the fruits, leaves, and blossoms of tomato plants, the cell numbers of *Salmonella* Newport declined significantly compared with the controls. More than 90% of the plants tested had no detectable levels of *Salmonella* by day 5 for blossoms (Allard et al., 2014).

Bacterial phytopathogens also affect enteric pathogens proliferation in produce. Supermarket produce surveys showed that 60% of produce showing symptoms of soft rot also harbored presumptive *Salmonella* (Brandl et al., 2013). *Salmonella* cells inoculated in pre-existing aggregates of *Pseudomonas syringae*, *Pseudomonas fluorescens*, and *Erwinia* spp. had a greater probability of surviving dry conditions on lettuce and cilantro leaves than as solitary cells (Poza-Carrion et al., 2013). Within soft rots, *Salmonella* reached population densities 10- to 100-fold higher than within intact plants (Cox et al., 2013). The current view is that enteric pathogens appear to be successful secondary colonists in post-harvest produce, benefiting from the action of phytopathogens, e.g., suppression of plant defense and plant tissue damage (lesions, water soaking, and soft rots) (Brandl et al., 2013). Therefore, the selection of cultivars resistant to phytopathogens would be important not only to increase yield and quality but also to support produce safety (Brandl et al., 2013; Bettini et al., 2016).

## Conclusion

The implementation of appropriate breeding practices could provide an additional important step toward produce safety. Currently the main picture is still in its infancy and it is difficult to provide directions on selecting safer cultivars or include them in risk assessments. Nevertheless, some data are useful: for example a study on tomato mutants showed that RIN gene, used to increase shelf life, has also showed some protection against enteric pathogens in ‘Ailsa Craig’ fruit. More studies must be done on different cultivars testing heterozygosity and alleles type (Marvasi et al., 2014a).

Excellent reviews also show that plants respond to *Salmonella* via defense pathways and that such genetic traits could also be used to achieve increase in produce safety (Teplitski et al., 2009; Brandl et al., 2013). At the same time, a better knowledge of the ecology of enteric pathogens in the environment, their interactions with plants and their persistence in wildlife are required to develop comprehensive solutions that build on the “One Health” concept.

Breeding practices have been used for a long time to reduce risk of plant pathogens. Can the same be done for pathogenic enteric pathogens? The question still remains extremely actual and open.

## Author Contributions

TH and MM focused on the microbiological component. AL and AB on the agronomical part. All authors wrote the manuscript.

## Conflict of Interest

The authors declare that the research was conducted in the absence of any commercial or financial relationships that could be construed as a potential conflict of interest.

## References

[B1] Allard S. Enurah A. Strain E. Millner P. Rideout S. L. Brown E. W. (2014). In situ evaluation of *Paenibacillus alve*i in reducing carriage of *Salmonella enterica* Serovar Newport on whole tomato plants. *Appl. Environ. Microbiol.* 80 3842–3849. 10.1128/AEM.00835-14 24747888 PMC4054204

[B2] Bai Y. Lindhout P. (2007). Domestication and breeding of tomatoes: what have we gained and what can we gain in the future? *Ann. Bot.* 100 1085–1094. 10.1093/aob/mcm150 17717024 PMC2759208

[B3] Barak J. D. Kramer L. C. Hao L. Y. (2011). Colonization of tomato plants by *Salmonella enterica* is cultivar dependent, and type trichomes are preferred colonization sites. *Appl. Environ. Microbiol.* 77 498–504. 10.1128/AEM.01661-10 21075871 PMC3020540

[B4] Barak J. D. Liang A. Narm K. E. (2008). Differential attachment to and subsequent contamination of agricultural crops by *Salmonella enterica*. *Appl. Environ. Microbiol.* 74 5568–5570. 10.1128/AEM.01077-08 18606796 PMC2546622

[B5] Barak J. D. Schroeder B. K. (2012). Interrelationships of food safety and plant pathology: the life cycle of human pathogens on plants. *Annu. Rev. Phytopathol.* 50 241–266. 10.1146/annurev-phyto-081211-172936 22656644

[B6] Bartz J. A. Marvasi M. Teplitsi M. (2014). “*Salmonella* and tomatoes,” in *The Produce Contamination Problem: Causes and Solution*, eds Matthews K. R. Sapers G. M. Gerba C. P. (Cambridge, MA: Academic Press), 269–289.

[B7] Bettini P. P. Marvasi M. Fani F. Lazzara L. Cosi E. Melani L. (2016). *Agrobacterium rhizogenes* rolB gene affects photosynthesis and chlorophyll content in transgenic tomato (*Solanum lycopersicum* L.) plants. *J. Plant Physiol*. 204 27–35. 10.1016/j.jplph.2016.07.010 27497742

[B8] Brandl M. T. Cox C. E. Teplitski M. (2013). *Salmonella* interactions with plants and their associated microbiota. *Phytopathology* 103 316–325. 10.1094/PHYTO-11-12-0295-RVW 23506360

[B9] Carrari F. Baxter C. Usadel B. Urbanczyk-Wochniak E. Zanor M. I. Nunes-Nesi A. (2006). Integrated analysis of metabolite and transcript levels reveals the metabolic shifts that underlie tomato fruit development and highlight regulatory aspects of metabolic network behavior. *Plant Physiol.* 142 1380–1396. 10.1104/pp.106.088534 17071647 PMC1676044

[B10] CDC (2019). *Web site Centers for Disease Control.* Available online at: https://www.cdc.gov/foodsafety/outbreaks/index.html (accessed December 20, 2019).

[B11] Cox C. E. McClelland M. Teplitski M. (2013). Consequences of disrupting *Salmonella* AI-2 signaling on interactions within soft rots. *Phytopathology* 103 352–361. 10.1094/PHYTO-09-12-0237-FI 23324045

[B12] Devleesschauwer B. Marvasi M. Giurcanu M. C. Hochmuth G. J. Speybroeck N. Havelaar A. H. (2017). High relative humidity pre-harvest reduces post-harvest proliferation of *Salmonella* in tomatoes. *Food Microbiol.* 66 55–63. 10.1016/j.fm.2017.04.003 28576373

[B13] Dewey-Mattia D. Manikonda K. Hall A. J. Wise M. E. Crowe S. J. (2018). Surveillance for foodborne disease outbreaks – United States, 2009–2015. *MMWR Surveill. Summ.* 67:1 10.15585/mmwr.ss6710a1 PMC6061962 30048426

[B14] Erickson M. C. Liao J. Y. Payton A. S. Cook P. W. Den Bakker H. C. Bautista J. (2019a). Survival of *Salmonella enterica* and *Escherichia coli* O157:H7 sprayed onto the foliage of field-grown cabbage plants. *J. Food Prot.* 82 479–485. 10.4315/0362-028X.JFP-18-326 30806554

[B15] Erickson M. C. Liao J. Y. Payton A. S. Cook P. W. Den Bakker H. C. Bautista J. (2019c). Pre-harvest internalization and surface survival of *Salmonella* and *Escherichia coli* O157:H7 sprayed onto different lettuce cultivars under field and growth chamber conditions. *Int. J. Food Microbiol.* 291 197–204. 10.1016/j.ijfoodmicro.2018.12.001 30551016

[B16] Erickson M. C. Liao J. Y. Payton A. S. Cook P. W. Ortega Y. R. (2019b). Survival and internalization of *Salmonella* and *Escherichia coli* O157:H7 sprayed onto different cabbage cultivars during cultivation in growth chambers. *J. Sci. Food Agric.* 99 3530–3537. 10.1002/jsfa.9573 30624787

[B17] Fujisawa M. Ito Y. (2013). The regulatory mechanism of fruit ripening revealed by analyses of direct targets of the tomato MADS-box transcription factor RIPENING INHIBITOR. *Plant Signal. Behav.* 8:e24357. 10.4161/psb.24357 23518588 PMC3907458

[B18] Han S. Micallef S. A. (2014). *Salmonella* Newport and Typhimurium colonization of fruit differs from leaves in various tomato cultivars. *J. Food Prot.* 77 1844–1850. 10.4315/0362-028X.JFP-13-562 25364916

[B19] Hubbard P. A. Liang X. Schulz H. Kim J. J. P. (2003). The crystal structure and reaction mechanism of *Escherichia coli* 2,4-dienoyl-CoA reductase. *J. Biol. Chem.* 278 37553–37560. 10.1074/jbc.M304642200 12840019

[B20] Iniguez A. L. Dong Y. Carter H. D. Ahmer B. M. M. Stone J. M. Triplett E. W. (2005). Regulation of enteric endophytic bacterial colonization by plant defenses. *Mol. Plant Microbe Interact.* 18 169–178. 10.1094/MPMI-18-0169 15720086

[B21] Jadhav S. Singh B. Salunkhe D. K. (1972). Metabolism of unsaturated fatty acids in tomato fruit: linoleic and linolenic acid as precursors of hexanal. *Plant Cell Physiol.* 13 449–459. 10.1093/oxfordjournals.pcp.a074758

[B22] Lapidot A. Yaron S. (2009). Transfer of *Salmonella enterica* Serovar Typhimurium from contaminated irrigation water to parsley is dependent on curli and cellulose, the biofilm matrix components. *J. Food Prot.* 72 618–623. 10.4315/0362-028X-72.3.618 19343953

[B23] Liu M. Pirrello J. Chervin C. Roustan J. P. Bouzayen M. (2015). Ethylene control of fruit ripening: revisiting the complex network of transcriptional regulation. *Plant Physiol.* 169 2380–2390. 10.1104/pp.15.01361 26511917 PMC4677914

[B24] Maruzani R. Sutton G. Nocerino P. Marvasi M. (2019). Exopolymeric substances (EPS) from *Salmonella enterica*: polymers, proteins and their interactions with plants and abiotic surfaces. *J. Microbiol.* 57 1–8. 10.1007/s12275-019-8353-y 30552630

[B25] Marvasi M. Cox C. E. Xu Y. Noel J. T. Giovannoni J. J. Teplitski M. (2013b). Differential regulation of *Salmonella* Typhimurium genes involved in O-antigen capsule production and their role in persistence within tomato fruit. *Mol. Plant Microbe Interact.* 26 793–800. 10.1094/MPMI-09-12-0208-R 23489058

[B26] Marvasi M. George A. S. Giurcanu M. Hochmuth G. J. Noel J. T. Gause E. (2014b). Effects of nitrogen and potassium fertilization on the susceptibility of tomatoes to post-harvest proliferation of *Salmonella enterica*. *Food Microbiol.* 43 20–27. 10.1016/j.fm.2014.03.017 24929878

[B27] Marvasi M. George A. S. Giurcanu M. C. Hochmuth G. J. Noel J. T. Teplitski M. (2015b). Effect of the irrigation regime on the susceptibility of pepper and tomato to post-harvest proliferation of *Salmonella enterica*. *Food Microbiol.* 46 139–144. 10.1016/j.fm.2014.07.014 25475277

[B28] Marvasi M. Hochmuth G. Teplitski M. (2015a). The role of crop production practices and weather conditions in microbiological safety of tomatoes and peppers. *Food Microbiol.* 46 139–144. 10.13140/RG.2.2.20981.01762 25475277

[B29] Marvasi M. Hochmuth G. J. Giurcanu M. C. George A. S. Noel J. T. Bartz J. (2013a). Factors that affect proliferation of *Salmonella* in tomatoes post-harvest: the roles of seasonal effects, irrigation regime, crop and pathogen genotype. *PLoS One* 8:e80871. 10.1371/journal.pone.0080871 24324640 PMC3851777

[B30] Marvasi M. Noel J. T. George A. S. Farias M. A. Jenkins K. T. Hochmuth G. (2014a). Ethylene signalling affects susceptibility of tomatoes to *Salmonella*. *Microb. Biotechnol.* 7 545–555. 10.1111/1751-7915.12130 24888884 PMC4265073

[B31] Moorman G. W. Gevens A. J. Granke L. H. Hausbeck M. K. Hendricks K. Roberts P. D. (2014). “Sources and distribution systems of irrigation water and their potential risks for crop health,” in *Biology, Detection, and Management of Plant Pathogens in Irrigation Water*, eds Hong C. Moorman G. W. Wohanka W. Buttner C. (St. Paul, MN: APS Press), 3–12. 10.1094/9780890544914.002

[B32] Murray K. Wu F. Shi J. Jun Xue S. Warriner K. (2017). Challenges in the microbiological food safety of fresh produce: limitations of post-harvest washing and the need for alternative interventions. *Food Qual. Saf.* 1 289–301. 10.1093/fqsafe/fyx027

[B33] Noel J. T. Arrach N. Alagely A. Mcclelland M. Teplitski M. (2010). Specific responses of *Salmonella enterica* to tomato varieties and fruit ripeness identified by *In Vivo* expression technology. *PLoS One* 5:e12406. 10.1371/journal.pone.0012406 20824208 PMC2930847

[B34] Osorio S. Alba R. Damasceno C. M. B. Lopez-Casado G. Lohse M. Zanor M. I. (2011). Systems biology of tomato fruit development: combined transcript, protein, and metabolite analysis of tomato transcription factor (nor, rin) and ethylene receptor (Nr) mutants reveals novel regulatory interactions. *Plant Physiol.* 157 405–425. 10.1104/pp.111.175463 21795583 PMC3165888

[B35] Poza-Carrion C. Suslow T. Lindow S. (2013). Resident bacteria on leaves enhance survival of immigrant cells of *Salmonella enterica*. *Phytopathology* 103 341–351. 10.1094/PHYTO-09-12-0221-FI 23506362

[B36] Singh P. Santoni S. Weber A. This P. Péros J. P. (2019). Understanding the phyllosphere microbiome assemblage in grape species (*Vitaceae*) with amplicon sequence data structures. *Sci. Rep.* 9:14294. 10.1038/s41598-019-50839-0 31586145 PMC6778096

[B37] Teplitski M. Barak J. D. Schneider K. R. (2009). Human enteric pathogens in produce: un-answered ecological questions with direct implications for food safety. *Curr. Opin. Biotechnol*. 20 166–171. 10.1016/j.copbio.2009.03.002 19349159

[B38] Teplitski M. de Moraes M. (2018). Of mice and men. and plants: comparative genomics of the dual lifestyles of enteric pathogens. *Trends Microbiol*. 26 748–754. 10.1016/j.tim.2018.02.008 29502873

[B39] Tokarskyy O. De J. Fatica M. K. Brecht J. Schneider K. R. (2018). Survival of *Escherichia coli* O157:H7 and *Salmonella* on bruised and unbruised tomatoes from three ripeness stages at two temperatures. *J. Food Protect*. 81 2028–2033. 10.4315/0362-028X.JFP-18-220 30481483

[B40] U.S. FDA (2019). *Investigation Summary: Factors Potentially Contributing to the Contamination of Romaine Lettuce Implicated in the Fall 2018 Multi-State Outbreak of E. coli* O157:H7 Silver Spring, ML: U.S. Food and Drug Administration.

[B41] USDA - Statistics (2019). *Annual Reports: Crop Acreage, Yields, Areas Harvested, and Other Production Information.* Available online at: https://www.usda.gov/topics/farming/crop-production (accessed December 21, 2019).

